# Unique phenotype in a patient with CHARGE syndrome

**DOI:** 10.1186/1687-9856-2011-11

**Published:** 2011-10-13

**Authors:** Shobhit Jain, Hyung-Goo Kim, Felicitas Lacbawan, Irene Meliciani, Wolfgang Wenzel, Ingo Kurth, Josefina Sharma, Morris Schoeneman, Svetlana Ten, Lawrence C Layman, Elka Jacobson-Dickman

**Affiliations:** 1 State University of New York Downstate Medical Center, Children's Hospital at Downstate, Department of Pediatrics, Division of Pediatric Endocrinology, Brooklyn, NY 11203 USA; 2 Institute of Molecular Medicine and Genetics, The Medical College of Georgia, Section of Reproductive Endocrinology, Infertility, and Genetics, Department of Obstetrics and Gynecology, Augusta, GA 30912, USA; 3 State University of New York Downstate Medical Center, Department of Pathology, Division of Molecular Pathology, Brooklyn, NY 11203, USA; 4 Institute of Nanotechnology, Karlsruhe Institute of Technology, PO Box 3640. 76021 Karlsruhe, Germany; 5 Institute of Human Genetics, University Hospital Jena Kollegiengasse 1007743 Jena, Germany; 6 State University of New York Downstate Medical Center, Children's Hospital at Downstate, Department of Pediatrics, Division of Pediatric Nephrology, Brooklyn, NY 11203 USA

## Abstract

CHARGE is a phenotypically heterogeneous autosomal dominant disorder recognized as a cohesive syndrome since the identification of *CHD7 *as a genetic etiology. Classic features include: Coloboma, Heart defects, Atresia choanae, Retarded growth and development, Genitourinary abnormalities, and Ear anomalies and/or deafness. With greater accessibility to genetic analysis, a wider spectrum of features are emerging, and overlap with disorders such as DiGeorge syndrome, Kallmann syndrome, and Hypoparathyroidism Sensorineural Deafness and Renal Disease syndrome, is increasingly evident. We present a patient with a unique manifestation of CHARGE syndrome, including primary hypoparathyroidism and a limb anomaly; to our knowledge, he is also the first CHARGE subject reported with bilateral multicystic dysplastic kidneys. Furthermore, with structural modeling and murine expression studies, we characterize a putative *CHD7 *G744S missense mutation. Our report continues to expand the CHARGE phenotype and highlights that stringent fulfillment of conventional criteria should not strictly guide genetic analysis.

## Introduction

The CHARGE syndrome (MIM 214800) is an autosomal dominant or sporadic disorder of variable multisystemic congenital anomalies that occurs with an incidence of approximately 1 in 10,000 [1,2]. Heterozygous *CHD7 *(chromodomain helicase DNA-binding protein 7, MIM 608892) mutations have been identified in approximately 60%-70% of patients with clinically diagnosed CHARGE Syndrome and are most commonly due to *de novo *truncating mutations. Furthermore, *CHD7 *mutations are reported throughout the entire coding sequence of the gene without an apparent pattern or cluster, and meaningful genotype-phenotype correlations have not been recognized [1-3].

The first descriptions of this syndrome were provided by Hall and Hitner independently in 1979 [4,5], though it was in 1981 that Pagan and colleagues coined the acronym CHARGE to summarize its dominant features: coloboma, heart defects, atresia choanae, retarded growth and development, genital and/or urinary abnormalities, ear anomalies and/or deafness [6]. It was only in 2004 that the *CHD7 *gene was established as a genetic etiology for CHARGE syndrome by Vissers et al [7]. A wider spectrum of associated features has since emerged, albeit less consistently, and includes hyposmia or anosmia, cleft lip and palate, hypocalcemia [8,9], and tracheoesophageal fistula [10]. As such, CHARGE Syndrome has several overlapping clinical characteristics with DiGeorge syndrome [11], Kallmann syndrome, and Hypoparathyroidism, Sensorineural Deafness, and Renal Disease (HDR) (Barakat's syndrome) [12,13]. As the CHARGE phenotype continues to expand, particularly into the clinical purview of other conditions, its diagnosis becomes more challenging as well as increasingly inclusive.

Herein, we report a patient with a unique presentation of CHARGE syndrome, including primary hypoparathyroidism, bilateral multicystic dysplastic kidneys (MCDK), and an atypical limb anomaly; he carried a *CHD7 *mutation that has not been previously characterized. Our report expands the spectrum of phenotypes associated with *CHD7 *mutations.

## Clinical Report

**Figure 1:**
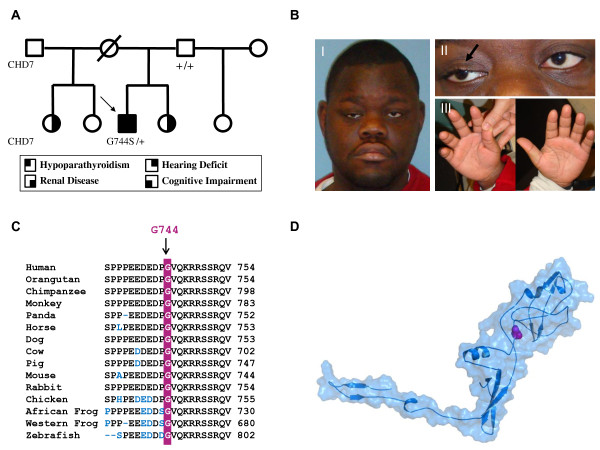
**A patient with a unique presentation of CHARGE syndrome and a G744S *CHD7 *mutation**. **A**. Pedigree of a CHARGE patient with a novel *CHD7 *mutation. circle: female; square: male; arrow: proband; +: wild type allele. **B**. Photographs of the facies (I), eyes (II), and hands (III) of the *CHD7 *G744S heterozygous proband. **C**. Evolutionary conservation of the residue Gly744. ClustalW multiple alignment of partial protein sequence of CHD7 orthologs. The position of residue G744 altered by one heterozygous nucleotide change is marked by arrow and red letters in the corresponding segment of the multiple alignment. The amino acid residues that differ from the sequence of the human CHD7 protein are indicated blue. Gly744 residue is evolutionarily fully conserved in all fifteen available CHD7 orthologs. **D**. Structural model of the amino acid regions spanning 651-794 of the CHD7 protein obtained by sequence homology to a bacterial flagellar filament. The site of the mutation (indicated in magenta), which is predicted to be detrimental by POLYPHEN, lies on a protein interaction surface as indicated by SSPIDER.

The proband, an 18 year old African American male, presented to us initially for management of refractory hypocalcemia. He was born weighing 795 grams at 25 weeks gestation to a 31-year old woman with severe hypertension, who died in the postpartum period of a myocardial infarction. In infancy, he underwent cardiac surgery for a ventricular septal defect, and another to correct a right eyelid coloboma. Additionally, he had congenital hypothyroidism, bilateral sensorineural hearing loss, and severe global developmental delay; he began walking during his third year of life, remains unable to independently dress or tie shoe laces, and he has a vocabulary of fewer than 5 words. At a young age, the family had been told that he had Down syndrome, a diagnosis he carried until our meeting. Our initial consultation for hypocalcemia was during a hospitalization for complications of end stage renal disease secondary to MCDK, which was diagnosed in early life based on X-ray computed tomography (CT) showing dysplastic kidneys with multiple cysts. Interestingly, both a full sister and maternal half sister had severe hearing deficits and renal disease (Figure 1A). Unfortunately, neither was available for further characterization.

His physical examination revealed an overweight and short young man (weight 65.9kg, height 150.5cm or -3.58 SDS, body mass index 29.3 kg/m^2^), communicating primarily with hand gestures including pointing. He had brachycephaly with a flat facial profile, short forehead and facial asymmetry, slightly upslanted eyes with mild right ptosis, a scar from eyelid coloboma corrective surgery, and mildly prominent ears with low nasal bridge, upturned nasal tip, and smooth philtrum (Figure 1B.I and 1B.II). He had no cleft lip or palate. The chest was symmetrical. He had scars from cardiac surgery on the left anterior and posterior walls, and on the anterior abdominal wall at the site of a neonatal gastric tube placement. On examination of the extremities, his palmar creases were normal and he had a flexion deformity of the right thumb whereby this digit was fixed in the adducted position (Figure 1B.III). His genital examination revealed Tanner V pubic hair, and bilaterally descended testes consistent in size with early to mid-pubertal range (5 mL right and 8 mL left, using Prader Orchidometer).

A CT scan of the head showed bilateral basal ganglia calcifications and scattered calcification in the frontal white matter and cerebellum, likely related to chronic hypcalcemia. Olfactory structures could not be evaluated. X-ray of his hands revealed no osseous abnormalities to explain his right thumb deformity.

The initial laboratory results included low serum calcium (6.3 mg/dL, normal 8.3-10 mg/dL) normal albumin (4.2 g/dL, normal 3.5-5.8 g/dL), low phosphorus (1.1 mg/dL, normal 2.5-5 mg/dL), normal 25-hydroxy vitamin D (38 ng/mL, normal 20-100 ng/mL), slightly low magnesium (1.6 mg/dL, normal 1.9-2.7 mg/dL) coincident with low parathyroid hormone level (4.9 pg/mL, normal 15-65 pg/mL), indicating primary hypoparathyroidism. His low phosphorus was due to phosphate wasting associated with polyuria of end stage renal disease (ESRD). He had normal complete blood cell counts with no evidence of white cell line depression. Additionally, despite having early to mid-pubertal sized testes, his LH, FSH and Testosterone were not in the hypogonadal range (LH 4.1 mIU/mL, FSH 6.8 mIU/mL, Testosterone 505 ng/dL), which is not consistent with Idiopathic Hypogonadotropic Hypogonadism.

In view of his dysmorphic features and questionable history of Down Syndrome (MIM 190685), a karyotype was obtained which showed a normal 46, XY configuration in 200 stimulated peripheral lymphocytes. Fluorescent in situ hybridization (FISH) for a chromosome 22q11.2 deletion, associated with DiGeorge Syndrome (MIM 188400), was negative. To exclude a Calcium-Sensing Receptor defect (MIM 601199), sequencing of the *CASR *gene was conducted of the coding regions and exon/intron boundaries and did not reveal a mutation. In light of the patient's constellation of hypoparathyroidism, renal disease, as well as deafness, we performed microarray and then sequencing analyses for mutations in *GATA3 *gene associated with the HDR syndrome (MIM 146255) [13], and none were found. Finally, *CHD7 *mutation analysis was performed on a genomic sample after PCR amplification of exons 2 to 38. The primers for all exons flanked the respective intron-exon junctions and direct sequencing was performed in both forward and backward directions using automated fluorescence di-deoxy sequencing methods. This revealed 6 heterozygous unclassified variants in the *CHD7 *gene including 2 missense and 4 silent changes (Table 1). Among the missense changes, a G744S (NP_060250) resulting from a c.2230G→A nucleotide change (NM-017780), was found to be conserved in available *CHD7 *orthologs (Figure 1C) and was not identified in 192 control subjects. The subject's asymptomatic father, the only family member available for genetic analysis, was found not to carry the G744S substitution. An informed consent for all genetic testing and for image publication was obtained from father and from the subject's legal guardian.

**Table 1:** Sequence Analysis Revealed Six Unclassified Variants in the *CHD7 *Gene

Nucleotide Change NM_017780	Amion Acid Change NP_060250	SNP ID*
Missense changes		
c.2230G→A	p.G744S	-
c.6478G→A	p.A2160T	rs61753399
Silent Changes		
c.309G→A	p.S103S	rs41272435
c.657C→T	p.G219G	-
c.2124T→C	p.S708S	-
c.7590A→G	p.K2530K	rs61742801

*Reported in the Single Nucleotide Polymorphism database dbSNP:http:www.ncbi.nlm.nih.gov//SNP

### Structural Modeling of the CHD7 Protein

The *G744S *mutation, which is located near chromodomain 1 in exon 4, was hypothesized to possess functional implications in the pathogenesis of CHARGE Syndrome. We constructed a structural model for the portion of the CHD7 protein containing the mutation based on homology to a bacterial flagellar filament (pdb-code 1UCU) [14] (See Figure 1D and additional file 1 for methods and alignment). Based on this model, SPPIDER [15] identified the site of the mutation as a possible binding site. G744 is located at the protein surface, where mutations are most likely to affect protein function and signaling compared to mutations in the protein interior. In agreement with this observation, the bioinformatics based approach POLYPHEN predicted a possible damaging effect of the mutation with a PISC SCORE: 1.58 (values below 0.5 are considered benign and values above 1.0 possibly/probably damaging) [16]. The PISC score contains a sequence based estimate of the accessibility of the mutation site, which is underestimated in comparison with the predictions of the structural model. Therefore, the sequence based PSIC score likely underestimates the damaging effect of our mutation.

### Chd7 Murine Expression Studies

**Figure 2:**
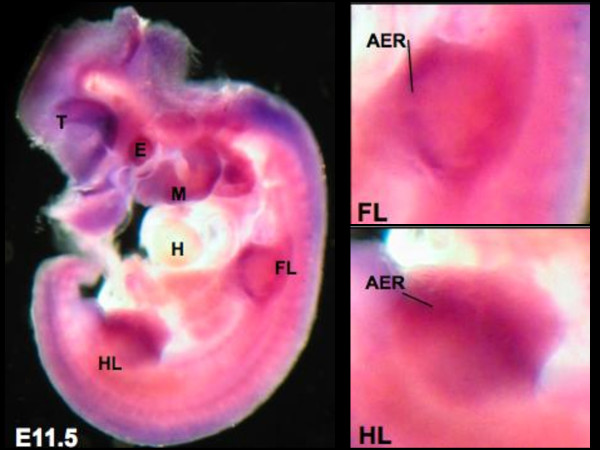
**Chd7 expression in the developing mouse**. At E11.5 *Chd7 *is broadly expressed and shows prominent signals with the *Chd7 *antisense probe in DIG labeled whole mount in-situ hybridization in the region of the distal tip of the limb buds (see also enlargements on the right). T: telencephalic vesicle, E: corneal ectoderm overlying lens vesicle, M: maxillary component of first branchial arch, H: heart, FL: forelimb, HL: hindlimb.

Throughout murine development *Chd7 *is broadly expressed, including organs classically anomalous in CHARGE Syndrome, such as the eyes, heart, and ears. To investigate the distribution of *Chd7 *transcripts in the developing limbs, we performed *in situ *hybridization analysis using whole mount preparations. The following primers were used for amplification of the probe spanning nucleotides 8305 to 9140 of the murine *Chd7 *transcript (NM_001081417): 5′-CAGGTGGCTGGAGGAGAACCC-3′ and 5′-CTTTACAGGGCCCTCCCTCGGCC-3′. Amplicons were ligated to a Topo-TA vector (Invitrogen) and subcloned into pBluescript via *Eco*RI and *Xba*I restriction sites. The probe was labeled with Digoxigenin (DIG) for whole-mount in situ hybridization following standard procedures. No specific signals were detected using the respective sense probes. By embryonic day E11.5, the limb buds are clearly divided into proximal and distal elements. By E12, the handplate showed evidence of angular contours at its peripheral margin, corresponding to the location of the future digits. At E12.5-E13 early evidence of digital rays that are separated by the digital interzones were apparent. We found high expression levels of *Chd7 *throughout these steps of limb development in DIG-labelled whole mount embryos. Pronounced *Chd7 *expression was noted at the limb bud apical ectodermal ridge (AER) (Figure 2), a thickened layer of ectodermal cells at the distal tip of the developing limb bud, which is a crucial organizing region during limb formation. This *Chd7 *expression pattern supports a role for this gene in limb development.

## Discussion

CHARGE is a phenotypically heterogeneous autosomal dominant disorder that has been recognized as a non-random cohesive syndrome since the identification of *CHD7 *mutations as an underlying etiology [1,2,7]. *CHD7*, at 8q12.1, encodes a protein of the chromodomain family [17,18]. The exact function of CHD7 has not been elucidated, however, in situ hybridization analysis during human development has demonstrated expression of this gene in the central nervous system, semicircular canals and the neural crest of the pharyngeal arches; thereby implicating the embryologic role of CHD7 in the development of the respective organs [19,20]. Furthermore, *CHD7 *mRNA expression was documented in the hypothalamus, pituitary and olfactory bulb in the rat and also demonstrated in both migratory and post-migratory GnRH neuronal cell lines [21]. Our subject carried a heterozygous G744S, not seen in controls; this missense change has been reported in one previous series and designated a polymorphism, though no functional studies were conducted and controls were not tested [22]. In this instance, the G744S change was seen in a family with three children with clinical CHARGE born to two different women and one man. A larger *CHD7 *rearrangement was additionally seen in all affected children, and found in mosaic form in the father's sperm cell DNA but not in lymphocytes. The G744S change, however, was found in two of the CHARGE children (the third was not tested) and in the completely unaffected father. Since the father was heterozygous and not mosaic for G744S, the authors considered G744S to be a non-pathogenic variant (Kohlhase J, unpublished data). *CHD7 *mutations have clinically variable expression, and clear genotype-phenotype correlations are not observed, even among patients with identical *CHD7 *mutations [1-3]. Asymptomatic carriers are reported as well, particularly in inherited forms [3]. We can speculate that the disparate putative effect of this mutation is subject to yet undefined secondary genetic, epigenetic or environmental influences; this has been demonstrated in other genetic disease models, such as idiopathic hypogonadotropic hypogonadism (IHH) and Kallmann syndrome [23-25]. Our bioinformatics based structural analysis, protein alignment, and DNA sequencing in normal controls, provide supportive evidence that the G744S is a true deleterious mutation involving a highly conserved amino acid, likely disrupting a crucial protein interaction site.

In this age of sophisticated and readily available genetic analysis, the diagnosis of CHARGE appears to be transforming from the rigid fulfillment of conglomerates of specific major and minor criteria, towards an approach more inclusive of patients with atypical or attenuated phenotypes, as was demonstrated by Kim et al [26]. CHARGE and Kallmann syndromes (KS, KAL5, MIM 612370), though distinct developmental disorders, were noted to share features of impaired olfaction and hypogonadism; thus *CHD7 *was hypothesized to be involved in the pathogenesis of KS even in the absence of the CHARGE phenotype. Among 197 patients, they identified seven heterozygous mutations (two splice and five missense, absent in ≥ 180 controls) in three sporadic KS and four sporadic normosmic IHH patients. Thus, sporadic *CHD7 *mutations occurred in 6% of IHH/KS patients studied, allowing them to conclude that IHH/KS can represent a milder allelic variant of CHARGE syndrome. Furthermore, Jongmans et al also identified de novo *CHD7 *mutations in 3 of 56 mixed KS and nIHH subjects. Interestingly, in retrospect, their IHH patients with *CHD7 *mutations had some CHARGE features, including colobomas, deafness, ear anomalies, cleft palate and short stature; however, not to the degree that would fulfill traditional criteria [27]. Supported by this rational, we can concluded that our subject, with an atypical eyelid coloboma, hearing loss, severe developmental delay, ventricular septal defect, short stature, and abnormal facies, in addition to other more recently described features, such as a limb anomaly, primary hypoparathyroidism and interrupted pubertal development, may be included in this designation.

CHARGE syndrome was initially not considered to involve the limb. Several subsequent reports have shown associated limb anomalies and one series reported at least one limb anomaly in over one third of 172 CHARGE patients; furthermore, there did not appear to be a common limb anomaly in their cohort and minor abnormalities were included [28]. Prasad et al in 1997 were among the first to report severe limb abnormalities, including camptodactyly, tibial hemimelia and severe club-foot, in a patient with clinical CHARGE Syndrome [29]. It is notable that "club-foot", or congenital talipes equinovarus, is typically not due to osseous malformation. In 2007, Van de Laar et al reported 3 patients with heterozygous *CHD7 *truncating mutations in distinct exons, who displayed several limb malformations, including tibial aplasia, monodactyly and bifid femora [30]. Other limb defects, including triphalangeal thumb, polydactyly of the foot, ectrodactyly, and radial aplasia, have been reported as well [1,19,29,31]. Sanlaville et al in 2006, studied expression of *CHD7 *during human embryonic development detecting a weak signal in limb bud mesenchym at C14 [20]. Our patient, added to those previously described, highlighted by our demonstration of strong *Chd7 *expression in murine limb buds, further supports the suggestion that limb abnormalities should be a more recognized feature within the phenotypic spectrum of CHARGE syndrome. Moreover, homology modeling revealed a similarity to a bacterial flagellae protein [14], which in turn has high homology with the human *TCN *gene, encoding the human cytoskeletal protein titin; in concert with actin, titin plays a dominant role in human cytoskeletal development. Interestingly, direct comparison of titin and 1UCU sequences revealed a 19 amino acid gap of highly charge amino acids (spanning aa 741-759, EDP**G**VQKRRSSRQVKRKRY), which are most likely involved in functional differences between the two proteins and hence confer particular vulnerability to functional changes upon mutation.

In CHARGE Syndrome, anomalies of the urinary tract are reported in 10-40%, and include neurogenic bladder, duplex kidneys, renal ectopia or agenesis, horseshoe kidneys, and ureteral anomalies. [1,32,33]. To our knowledge, our patient is the first CHARGE patient to be reported with the severe phenotype of MCDK, requiring renal replacement therapy with dialysis followed by transplant. Cystic renal dysplasia is an anomaly of differentiation of the fetal kidney, whereby the kidney contains primitive ducts and non-renal tissues such as cartilage, fat, hematopoietic tissue, and often cysts. The most severe form of cystic renal dysplasia is MCDK, and most cases are unilateral. In subjects with CHARGE, only few reports of simple renal cysts exist [1,2,30]. Interestingly, one CHARGE subject with a unilateral right-sided dysplastic kidney also had significant limb anomalies, including right tibia aplasia, left tibia hypoplasia, and bilateral club feet [30]. The molecular events involved in multicystic dysplastic kidneys, in general, remain to be elucidated, though studies have suggested involvement of *WNT-1 *[34], *FGFR3 *[35], and *PAX2 *[36]. The *PAX2 *gene is associated with the renal coloboma syndrome (MIM 120330), a syndrome characterized by renal hypoplasia and insufficiency, vesicoureteric reflux, and optic disc coloboma. Interestingly, multicystic dysplastic kidney has been reported in one family with Renal Coloboma Syndrome [36]. In a study of the distribution pattern of the *PAX2 *gene in human embryos, Tellier et al demonstrated that *PAX2 *gene expression occurs in the primordia affected with CHARGE syndrome. Therefore, *PAX2 *was further analyzed in 34 patients fulfilling the diagnostic criteria of the CHARGE syndrome, though no deletions or nucleotide variations of the coding sequence were detected, suggesting that mutations of the *PAX2 *gene was not a cause of the CHARGE [37]. Considering the embryonic expression of *PAX2 *reported, and the common clinical features of Renal Coloboma Syndrome with CHARGE, one can hypothesize that *CHD7 *may have a role in regulating *PAX2 *gene and therefore this overlapping pathway might be explored in CHARGE etiology, and perhaps contributes to the variable expression observed.

The significant clinical overlap and inherently variable features of CHARGE and DiGeorge Syndromes can make differentiating these initial diagnoses particularly challenging. Hypocalcemia has been reported in CHARGE, though hypoparathyroidism, specifically, has been implicated in only few cases [38,39]. A study comparing 25 CHARGE subjects with *CHD7 *mutations to a large cohort of subjects with 22q11.2 deletion syndrome, noted that features found more commonly in CHARGE syndrome included coloboma, choanal atresia, facial nerve palsy, tracheoesophageal fistula, and genital hypoplasia in boys. Interestingly, a high incidence of marked hypocalcemia was observed in their CHARGE study group (72%), and a pronounced spectrum of cell-mediated immunodeficiency ranging from lymphopenia (60%) to severe combined immunodeficiency (8%), was seen as well. Defects in humoral immunity were documented in 4 CHARGE patients and included severe hypogammaglobulinemia with decreased T-cell numbers, transient hypogammaglobulinemia during infancy, and immunoglobulin A deficiency [40]. An accurate distinction between these two entities can, therefore, be challenging but will influence genetic counseling; *CHD7 *mutations more typically occur sporadically, whereas 22q11.2 deletions are familial in 10% of cases [2,41].

## Conclusion

In summary, we report an 18 year old male with CHARGE syndrome and a unique phenotype, including primary hypoparathyroidism, bilateral MCDK, a limb anomaly, disrupted testicular growth, and an atypical eyelid coloboma, who harbored a heterozygous G744S *CHD7 *mutation. Our case emphasizes that CHARGE features are perhaps even more heterogeneous than previously described and should include limb anomalies more universally. Additionally, the stringent fulfillment of the conventional CHARGE criteria should not strictly guide genetic analysis. Furthermore, our report highlights that the clinical overlap of CHARGE with DiGeorge, HDR, and Kallmann Syndromes can pose a diagnostic challenge to the clinician, but the correct designation can have a critical impact on treatment, anticipatory guidance, and genetic counseling.

## Competing interests

The authors declare that they have no competing interests.

## Authors' contributions

SJ lead and participated in the phenotyping and genotyping of our proband and in the characterization of the pedigree members; he also contributed to writing the manuscript. HK and LL lead the characterization of the *CHD7 *G744S mutation. HK also contributed to writing this manuscript. IM and WW performed the structural modeling of the CHD7 Protein. FL and ST guided the phenotyping and genotyping of the proband. IK conducted the Chd7 murine expression studies. JS and MS conducted phenotyping of the proband's renal pathology. EJD conceived of this study, oversaw the phenotyping and genotyping of the proband and his family, and was the supervising writer of this manuscript. All authors read and approved the final manuscript.

## Supplementary Material

Additional file 1 **Additional file **1**includes further elaboration of *chd7 *protein modeling and alignement methods, as well as one figure of the protein sequence alignement**. Click here for file
