# Supplementary material for: Unique phenotype in a patient with CHARGE syndrome
Source: Int J Pediatr Endocrinol. 2011 Oct 13;2011(1):11. doi: 10.1186/1687-9856-2011-11 (PMC3216247; doi:10.1186/1687-9856-2011-11)
Supplement: Additional file 1 — includes further elaboration of chd7 protein modeling and alignement methods, as well as one figure of the protein sequence alignement. [file 1687-9856-2011-11-S1.DOC]

**ADDITIONAL FILE**

**Title:** CHD7 structural modeling based on homology to a bacterial flagellar filament
**Description:** Methods for structural modeling and alignment for the portion of the CHD7 protein containing the G744S mutation based on homology to a bacterial flagellar filament

An initial template selection was performed using the 3-D jury [1], Phyre [2] and Fugue [3] servers which failed to find high-homology templates for the whole protein. Because of the importance of having a model on the region of the mutation G744S, we focused our work on the region from 651 to 794 where a suitably high-homology template (1UCU) was predicted by Phyre. We used ClustalW to generate an alignment between CHD7 and 1UCU and used MOE to generate a model on the basis of the alignment. The sequence alignment of the region amino acids 601-794 obtained by alignment to 1UCU using ClustalW (4) is illustrated in Figure S1.


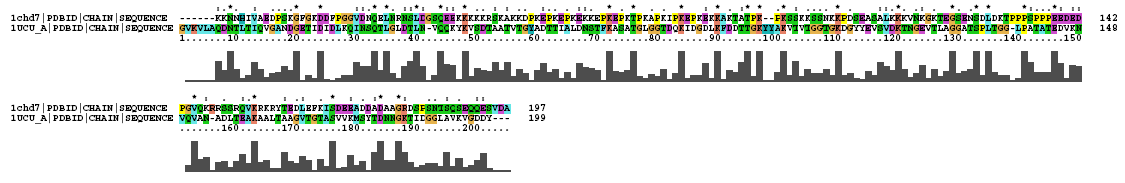


**Figure S1: comparison of the amino acid sequences from aa 601- 794 of CHD7 aligned with 1UCU. Sequence is colored by assigning a color to specific residues.**

**Stars indicate identity in all sequences, and dots indicate conserved types of amino acids.**

[1] Ginalski K., Elofsson A., Fischer D., Rychlewski L.. (2003)

*"3D-Jury: a simple approach to improve protein structure predictions."*

Bioinformatics. 19(8):1015-8

[2] Kelley L.A., Sternberg M.J.E. (2009)

Protein structure prediction on the web: a case study using the Phyre server.
Nature Protocols. 4, 363 - 371

[3]Shi J., Blundell T.L., Mizuguchi K (2001).

FUGUE: sequence-structure homology recognition using environment-specific substitution tables and structure-dependent gap penalties.

J Mol Biol. 310(1):243-57.

[4] K.B. Li (2003)

*ClustalW-MPI: ClustalW analysis using distributed and parallel computing.*

Bioinformatics, 19 (12) :1585-6
